# Icariside II protects against neuronal injury by modulating PI3K/AKT-dependent mitochondrial dynamics and apoptosis in Alzheimer’s disease models

**DOI:** 10.3389/fphar.2026.1911518

**Published:** 2026-09-21

**Authors:** Si-Yu Shan, Jia-Ming Bai, Yu Tian, Yu-Feng Shen, Ye-Xin Zhang, Yu-Han Cheng, Yang Liu, Hong-Yang Ning, Dong-Yu Min, Lu Ren, Hong-He Xiao

**Affiliations:** 1 School of Pharmacy, Liaoning University of Traditional Chinese Medicine, Dalian, China; 2 Traditional Chinese Medicine Experimental Center, Affiliated Hospital of Liaoning University of Traditional Chinese Medicine, Shenyang, China; 3 Mental Disorders Research Laboratory, Liaoning University of Traditional Chinese Medicine, Shenyang, China

**Keywords:** Alzheimer’s disease, apoptosis, Icariside II, mitochondrial dynamics, PI3K/AKT signaling pathway

## Abstract

**Introduction:**

Icariside II (ICS II), a major bioactive flavonoid metabolite from *Epimedium*, has been reported to exert neuroprotective effects, but its role in regulating mitochondrial dysfunction-related neuronal injury in Alzheimer’s disease (AD) has not been fully clarified.

**Methods:**

In this study, we administered ICS II to APP/PS1 transgenic mice and to two neuronal cell models of AD-like injury (Aβ-exposed HT22 cells and APP-overexpressing N2a cells). We evaluated cognitive behaviour, histopathological changes, Aβ burden, neuronal apoptosis, mitochondrial membrane potential (ΔΨm), mitochondrial reactive oxygen species (mtROS) levels, mitochondrial morphology and dynamics (including fragmentation, mitofusin-2 (Mfn2) expression, and dynamin-related protein 1 (Drp1) phosphorylation), as well as cytochrome c (Cyt c) release and caspase-3 activity. RNA-sequencing analysis of hippocampal tissue and pharmacological inhibition with the PI3K inhibitor LY294002 were employed to probe the involvement of the PI3K/AKT signalling pathway.

**Results and discussion:**

*In vivo*, ICS II improved cognitive performance, ameliorated hippocampal and cortical histopathological damage, decreased Aβ immunoreactivity, and reduced neuronal apoptosis. RNA-sequencing analysis suggested the involvement of PI3K/AKT signaling in the response to ICS II treatment. *In vitro*, ICS II increased cell viability, reduced oxidative stress, and inhibited apoptosis. These effects were associated with increased PI3K expression and AKT phosphorylation, restoration of ΔΨm, reduced mtROS accumulation, restored mitochondrial morphology (e.g., alleviated fragmentation, reduced punctate fragments, and increased mitochondrial length), decreased Cyt c release, increased Mfn2 expression, and decreased Drp1 phosphorylation at Ser616 and caspase-3 levels. Notably, co-treatment with LY294002 largely abolished these protective effects, indicating that PI3K/AKT signaling is functionally involved in this process. Taken together, our findings suggest that ICS II protects against AD-related neuronal injury are closely associated with PI3K/AKT-dependent regulation of mitochondrial dynamics and apoptosis, thereby providing a mechanistic foundation for further exploration of ICS II as a natural product-derived candidate for AD intervention.

## Graphical Abstract

**Figure:**
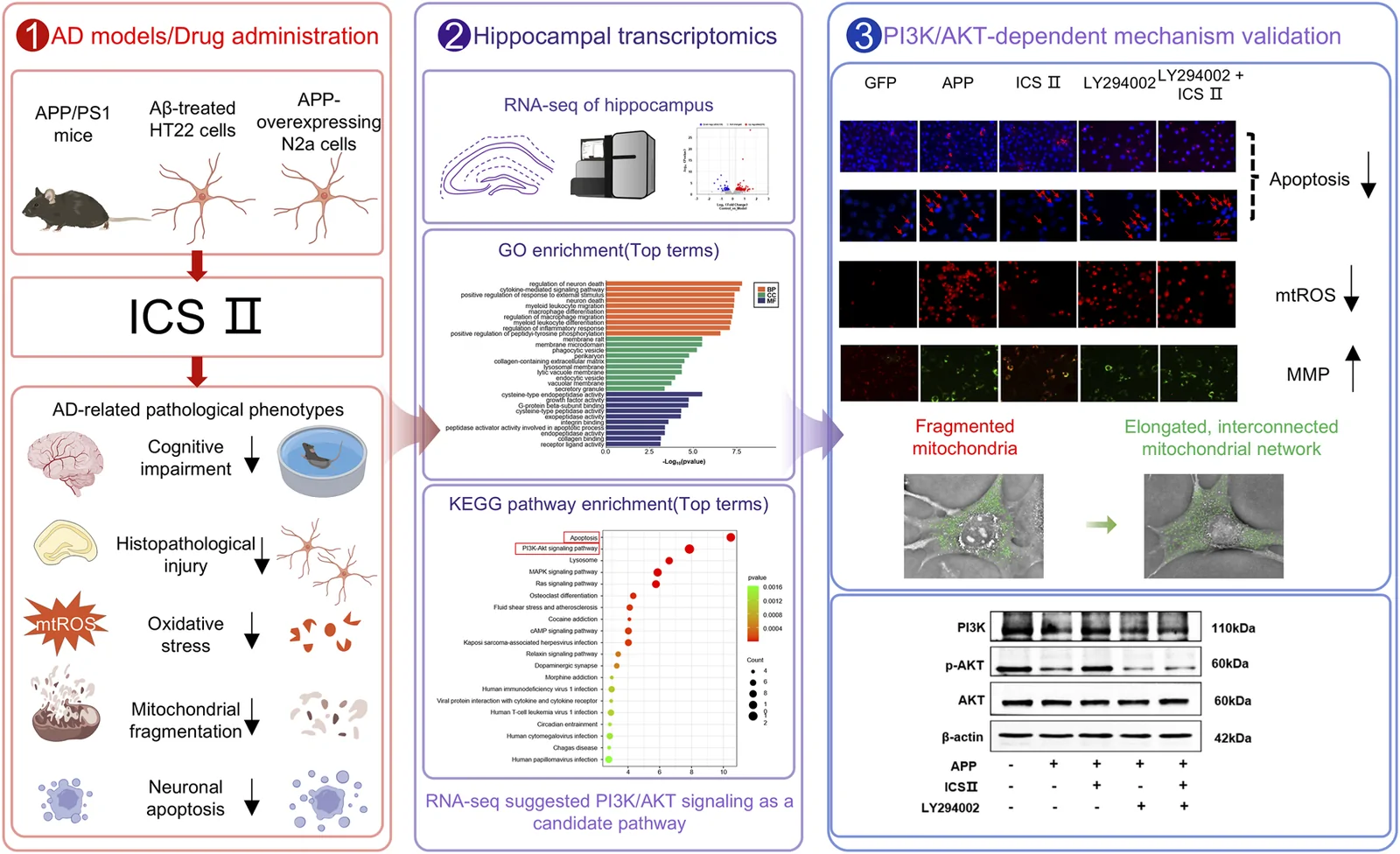

## 1 Introduction

Alzheimer’s disease (AD) is a progressive neurodegenerative disorder and the leading cause of dementia worldwide (Botella Lucena and Heneka, 2024; Zhang J. et al., 2024). Clinically, AD is characterized by a gradual decline in memory, cognition, executive function, and behavioral regulation, imposing a substantial and growing burden on patients, caregivers, and healthcare systems (Morrone et al., 2020; Oliver and Reddy, 2019). With global population aging, the prevalence of dementia is projected to increase dramatically in the coming decades, underscoring the urgent need for effective therapeutic strategies (Knopman et al., 2021). Despite extensive research efforts, currently available treatments provide only limited symptomatic benefit and do not effectively halt disease progression.

The pathological hallmarks of AD include extracellular deposition of β-amyloid (Aβ) plaques and intracellular neurofibrillary tangles composed of hyperphosphorylated Tau (Jack et al., 2018; Zheng and Wang, 2025). Beyond these classical features, accumulating evidence indicates that mitochondrial dysfunction is a central event in AD pathogenesis (Blagov et al., 2022; Thakral et al., 2023). As key regulators of ATP production, redox balance, calcium homeostasis, and apoptotic signaling, mitochondria are essential for neuronal survival and function (Calvo-Rodriguez and Bacskai, 2021). In neurons, mitochondrial integrity depends on the dynamic balance between fission and fusion (Flannery and Trushina, 2019). Increasing evidence suggests that this balance is disrupted early in AD, leading to excessive mitochondrial fragmentation, loss of mitochondrial membrane potential (ΔΨm), oxidative stress, impaired bioenergetics, and activation of apoptotic pathways (Bera et al., 2022; Flannery and Trushina, 2019; Reddy et al., 2012; Swerdlow et al., 2014; Swerdlow and Khan, 2004; Woo et al., 2021). Accordingly, modulation of mitochondrial quality control and mitochondrial dynamics has emerged as a promising therapeutic strategy for AD (Batista et al., 2021; Perez Ortiz and Swerdlow, 2019; Wang et al., 2021; Wang et al., 2020; Wang et al., 2019).

Current pharmacological therapies for AD remain inadequate. Cholinesterase inhibitors, including donepezil, rivastigmine, and galantamine, as well as the NMDA receptor antagonist memantine, provide only modest symptomatic relief and do not prevent progressive neurodegeneration (Burns et al., 2022; Cui et al., 2019; Hajjo et al., 2022; Marco-Contelles et al., 2006; Matsunaga et al., 2018; Rösler et al., 1999). Although amyloid-targeting monoclonal antibodies have recently expanded the therapeutic landscape, their overall clinical benefit remains limited and safety concerns, such as amyloid-related imaging abnormalities, continue to constrain their application. These limitations highlight the need for multi-target agents capable of intervening in key pathogenic processes beyond symptomatic control.

Natural products represent an important source of bioactive compounds for neuroprotective drug discovery. *Epimedium*, a traditional medicinal herb, contains multiple prenylated flavonoids with diverse pharmacological activities. Among them, Icariside II (ICS II), a major active metabolite generated from *Epimedium* flavonoid glycosides after oral administration, has attracted increasing attention because of its antioxidant, anti-inflammatory, anti-apoptotic, and neurotrophic properties (Singh and Singh, 2025). Previous studies have shown that ICS II attenuates neuronal injury in models of cerebral ischemia (Gao et al., 2023), inhibits hippocampal neuronal apoptosis (He et al., 2018), and enhances the expression of neurotrophic factors such as brain-derived neurotrophic factor (Liu et al., 2018). These findings suggest that ICS II may modulate multiple pathological processes relevant to neurodegeneration.

Previous studies further support the neuroprotective potential of ICS II in AD-related models. *Epimedium*-containing preparations improved learning and memory, reduced neuronal apoptosis, and promoted endogenous neurogenesis in experimental models (Xiao et al., 2020). In APP/PS1 transgenic mice, ICS II significantly enhanced hippocampal neurogenesis and improved cognitive performance, and these effects were associated with amelioration of mitochondrial dysfunction (Bai et al., 2025). In addition, ICS II promoted endogenous neural regeneration through activation of Wnt/β-catenin signaling (Xiao et al., 2022; Xiao et al., 2021). Collectively, these findings indicate that ICS II exerts pleiotropic neuroprotective effects and may influence neuronal survival, mitochondrial homeostasis, and regenerative responses in AD.

However, the precise mechanisms by which ICS II protects against AD-related neuronal injury remain incompletely understood. In particular, whether ICS II directly modulates mitochondrial dynamics and how such effects are linked to upstream survival signaling pathways have not been systematically clarified. Given the close relationship between PI3K/AKT signaling, mitochondrial homeostasis, and apoptosis, elucidating the role of this pathway may provide important mechanistic insight into the actions of ICS II.

Therefore, in the present study, we combined APP/PS1 transgenic mice with AD-related neuronal cell models and integrated RNA-sequencing analysis with biochemical and functional assays to investigate the protective effects of ICS II and the underlying mechanisms. Specifically, we examined whether ICS II alleviates neuronal injury by regulating PI3K/AKT-dependent mitochondrial dynamics and apoptosis. This study aims to expand the mechanistic basis of ICS II neuroprotection and to provide experimental support for its further development as a natural lead compound for AD-related neurodegeneration.

## 2 Materials and methods

### 2.1 Reagents and antibodies

ICS II (Lot No. wkq19050410, purity ≥98%) was obtained from the Weikeqi Biotech (Chengdu, China). The Cell Counting Kit-8 (CCK-8, G021-1), lactate dehydrogenase (LDH) assay kit (A020-2-2), total superoxide dismutase (SOD) assay kit (A001-1-2), and reduced glutathione (GSH) assay kit (A006-2-1) were purchased from Nanjing Jiancheng Bioengineering Institute (Nanjing, China). The Hoechst 33258 staining solution (WLA036a) was purchased from Wanlei Biotechnology (Shenyang, China). The mitochondrial membrane potential assay kit (JC-1, C7066) was purchased from Bioss Biotechnology (Beijing, China). The SF488 TUNEL Cell Apoptosis Detection Kit (T2196) and Mitochondrial Superoxide Assy Kit with MitoSO Red (CA1070) were from Beijing Solarbio Science and Technology Co., Ltd. (China). The TUNEL BrightRed Apoptosis Detection Kit (A113) was purchased from Vazyme (Nanjing, China).

Primary antibodies included: rabbit anti-B-cell lymphoma-2 (Bcl-2, WL03847), rabbit anti-Bcl-2-associated X protein (Bax, WL01637), rabbit anti-cysteinyl aspartate-specific proteinase-3 (Caspase-3, WL02117), rabbit anti-cytochrome c (Cyt c, WL02410), rabbit anti-phosphatidylinositol-3-kinase (PI3K, WL03380), rabbit anti-protein kinase B (AKT, WL003b), and rabbit anti-phosphorylated AKT (Ser473) (p-AKT, WLP0001a), all from Wanlei Biotechnology; rabbit anti-dynamin-related protein 1 (Drp1, bs-4100R) and rabbit anti-mitofusin 2 (Mfn2, bs-2988R) from Bioss Biotechnology; rabbit anti-phosphorylated Drp1 (Ser616) (p-Drp1, AF8470) from Qinke Biological Research Center Co., Ltd. (Jiangsu, China); and mouse anti-cleaved caspase-3 (68773-1-Ig), rabbit anti-NeuN (26975-1-AP) and mouse anti-APP (60342-1-Ig) from Proteintech (Wuhan, China).

### 2.2 Experimental animals

Six-month-old male APP/PS1 transgenic mice and age-matched C57BL/6 male mice (SPF grade) were purchased from Jiangsu Huachuang Xin Nuo Pharmaceutical Technology Co., Ltd. (SCXK [Su] 2021-0009). The animals were housed in a controlled environment with a temperature maintained at 23 °C–25 °C, relative humidity of 40%–60%, and a 12-h light/dark cycle.

All experiments were approved by the Institutional Animal Care and Use Committee (IACUC) of the affiliated institution and were conducted in accordance with the ARRIVE guidelines.

### 2.3 Animal grouping and drug administration

APP/PS1 mice were randomly divided into three groups (n = 10 per group): Model, ICS II, and Donepezil. Age-matched male wild-type C57BL/6J littermates were used as the normal control group (Control, n = 10). Mice in the ICS II group received ICS II at 10 mg/kg, and mice in the Donepezil group received donepezil at 0.65 mg/kg as a positive control. Both drugs were suspended in sodium carboxymethylcellulose (CMC-Na) and administered by oral gavage once daily for 28 consecutive days at a gavage volume of 10 mL/kg. Mice in the Control and Model groups received an equal volume of CMC-Na vehicle by oral gavage for the same duration. The doses of ICS II and donepezil were selected based on previous studies (Xiao et al., 2020; Xiao et al., 2022) (Figure 1A).

**FIGURE 1:**
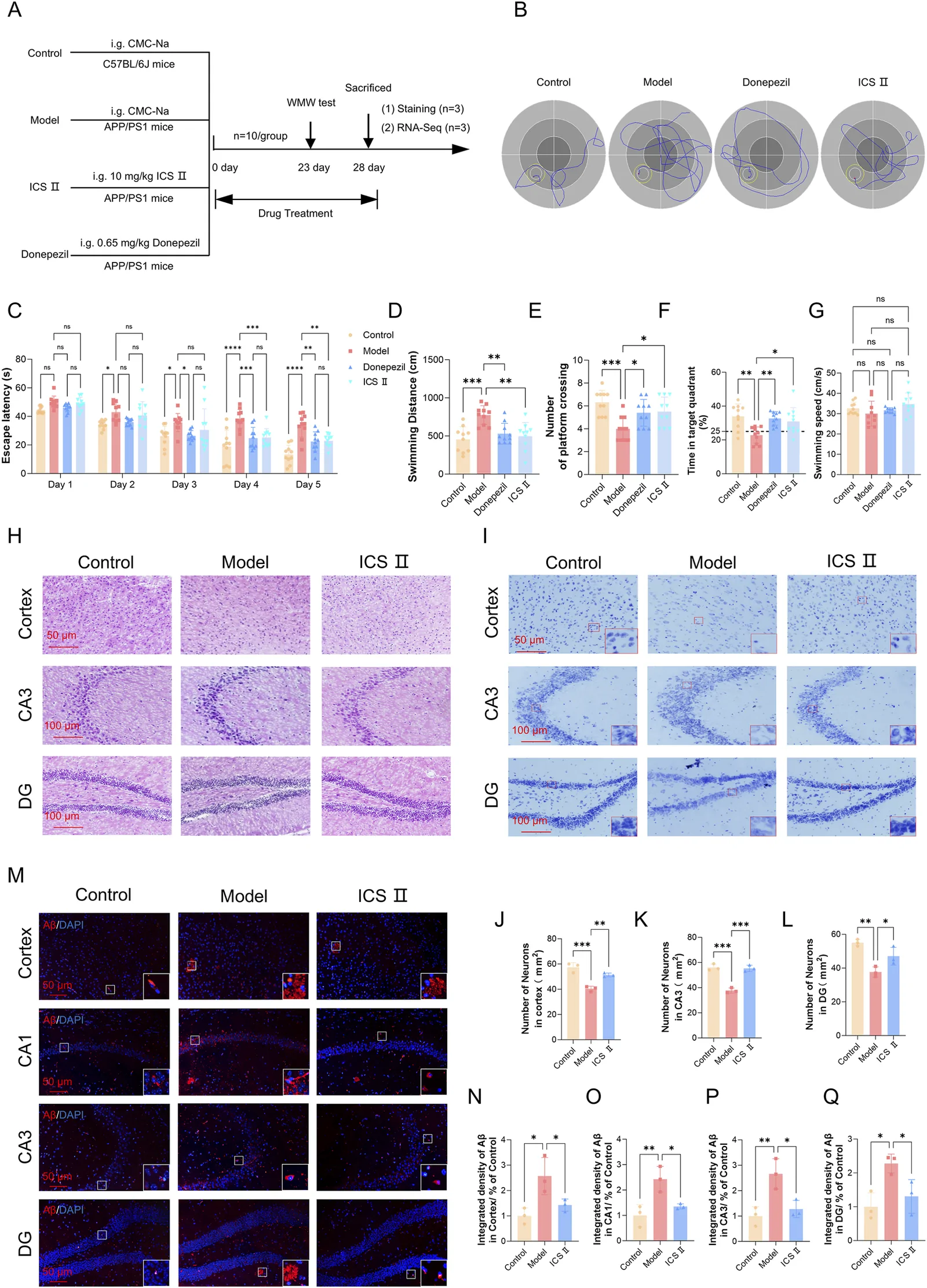
ICS II ameliorates cognitive deficits and histopathological damage in APP/PS1 mice. **(A)** An experiment outline for experimental design employed during the course of behavioral testing *in vivo*. **(B)** Representative swimming trajectories on day 5 of the navigation test. **(C)** Escape latency during the navigation test. **(D)** Swimming distance before first reaching the platform. **(E)** Number of platform crossings. **(F)** Percentage of time spent in the target quadrant; The dashed line indicates the chance exploration level (25%). **(G)** Swimming speed. **(H)** Representative images of H&E staining (cortex: scale bar = 100 μm; CA3, DG: scale bar = 50 μm). **(I)** Representative images of Nissl staining (cortex: scale bar = 100 μm; CA3, DG: scale bar = 50 μm). The boxed regions are magnified in the corresponding insets to show detailed morphology. **(J–L)** Quantitative analysis of Nissl bodies in the cortex **(J)** hippocampal CA3 **(K)** and DG **(L)** regions. **(M)** Representative images of Aβ peptides and DAPI immunofluorescence in the cerebral cortex and hippocampal regions. Scale bars = 50 μm. **(N–Q)** Semi-quantification of the fluorescence intensity in the cerebral cortex **(N)** hippocampal CA1 **(O)** CA3 **(P)** and DG **(Q)** regions. Statistical significance: *P < 0.05; **P < 0.01; ***P < 0.001; ****P < 0.0001. Data are presented as mean ± SD (Morris Water Maze Test, n = 10 mice/group; H&E, Nissl staining, Immunostaining, n = 3 mice/group).

### 2.4 Morris water maze (MWM) test

The MWM test was conducted over 6 days, comprising a 5-day navigation trial (days 23–27) followed by a probe trial on day 28. During navigation trials, mice underwent 4 trials per day. In each trial, they were placed into the water (maintained at 20 °C–21 °C) from fixed starting points (Chen et al., 2025). Each trial lasted a maximum of 60 s, and the escape latency was recorded. If a mouse failed to find the hidden platform within 60 s, it was gently guided to the platform and allowed to stay for 20 s (Zhou et al., 2025). For the probe trial, the platform was removed, and mice were released from the opposite quadrant. Swimming trajectories were tracked for 60 s, and the time spent and number of crossings in the target quadrant were recorded to evaluate spatial memory (n = 10 mice/group).

### 2.5 Brain tissue collection

After the MWM test, three mice were randomly selected from each group and anesthetized by intraperitoneal injection of sodium pentobarbital. The mice were then transcardially perfused with ice-cold PBS, followed by 4% paraformaldehyde (PFA). Brains were rapidly removed and post-fixed in 4% PFA overnight at 4 °C, followed by dehydration in 30% sucrose solution overnight at 4 °C. The brain tissues were then embedded in optimal cutting temperature (OCT) compound, and 10-μm-thick coronal sections were prepared using a cryostat (Leica CM1950 S). The sections were stored at −20 °C until further use.

For RNA sequencing, three additional mice from each group were anesthetized as described above. Hippocampal tissues were rapidly dissected on ice, immediately frozen in liquid nitrogen, and stored at −80 °C until subsequent analysis.

### 2.6 Hematoxylin-eosin (H&E) and Nissl staining

Brain sections were allowed to equilibrate to room temperature for 20 min, then fixed in 4% PFA. For H&E staining, sections were stained with hematoxylin for 5 min, rinsed, differentiated, blued in 0.5% ammonia water, counterstained with eosin for 3–5 s, dehydrated through graded ethanol, cleared in xylene, and mounted (n = 3 mice/group). Nissl staining was performed at 40 °C for 5 min in Nissl staining solution, followed by ethanol dehydration, xylene clearing, and mounting (n = 3 mice/group). Bright-field images of the cortex and hippocampal CA3, and DG regions were captured using a fluorescence microscope (Nikon Ti-S). Quantitative analysis of Nissl-positive neurons is detailed in Supplementary Material.

### 2.7 TUNEL staining

Apoptosis of hippocampal neurons was assessed using the Terminal deoxynucleotidyl transferase dUTP nick end labeling (TUNEL) assay. After equilibration at room temperature and permeabilization with 0.5% Triton X-100, sections were washed with PBS and incubated in 50 μL TUNEL reaction mixture (prepared per kit instructions) at 37 °C for 1 h. After washing, sections were counterstained with DAPI at room temperature for 5 min. Images were acquired from the same predefined areas of the cortex and hippocampal CA1, CA3, and dentate gyrus regions using a Nikon Ti-S fluorescence microscope under identical imaging conditions. TUNEL-positive cells were quantified using ImageJ software by an investigator blinded to the experimental groups (n = 3 mice per group).

### 2.8 Immunofluorescence staining

After frozen sections were rewarmed to room temperature, they were washed with PBS. The sections were then fixed with 4% PFA. After permeabilization with 0.5% Triton X-100 and blocking with 5% bovine serum albumin (BSA), the sections were incubated overnight at 4 °C with the following primary antibodies: rabbit anti-Aβ_1-42_ (1:100), rabbit anti-NeuN (1:200), and mouse anti-cleaved caspase-3 (1:500). The next day, the sections were incubated with corresponding fluorescent secondary antibodies at room temperature for 2 h in the dark: Alexa Fluor 488-conjugated goat anti-rabbit (1:100), Cy3-conjugated goat anti-mouse (1:150) and Cy3-conjugated donkey anti-rabbit (1:100). Subsequently, the sections were washed with PBS, mounted with an antifade mounting medium containing DAPI. Images were acquired from the same predefined cortical and hippocampal CA1, CA3, and dentate gyrus regions under identical imaging conditions. ImageJ software was used for blinded image analysis. Aβ staining was quantified as the mean fluorescence intensity within the predefined region of interest after background subtraction, whereas NeuN- and cleaved caspase-3-positive cells were counted using consistent fluorescence thresholds (n = 3 mice per group).

### 2.9 RNA-sequencing analysis

The total RNA was extracted using TRIzol, and mRNA libraries were constructed using the NEBNext Ultra II RNA Library Prep Kit for Illumina and sequenced on an Illumina platform. Raw reads were quality-controlled using FastQC v0.11.8, filtered to remove adapters and low-quality reads, aligned to the mouse reference genome using HISAT2 v2.0.5, and quantified using HTSeq v0.9.1. Differential expression analysis was performed using DESeq v1.20.0 based on raw read counts. Genes with |log_2_ (foldchange)| > 0.263 and *P* < 0.05 were defined as differentially expressed genes (DEGs) (Durocher et al., 2019; Zhang et al., 2019). The biological significance of the DEGs was assessed using the Gene Ontology (GO) and Kyoto Encyclopedia of Genes and Genomes (KEGG) databases to determine the associated biological processes. GO terms and KEGG pathways with *P* < 0.05 were considered significantly enriched (Cui et al., 2024; Mao et al., 2024). Three biological replicates were used per group (n = 3).

### 2.10 Establishment of Aβ-HT22 and APP-N2a cell models

HT22 cells were cultured in high-glucose Dulbecco’s Modified Eagle Medium (H-DMEM) supplemented with 10% fetal bovine serum (FBS) and 1% penicillin/streptomycin (P/S) at 37 °C in a humidified incubator containing 5% CO_2_. For model establishment, HT22 cells were seeded into 96-well plates at 5 × 10^3^ cells/well. After adherence, cells were treated with Aβ_1-42_ at concentrations of 0, 5, 10, 15, 20, or 40 μM for 24 h, and viability was assessed via CCK-8 to determine the optimal modeling concentration. Cells were then exposed to this optimal concentration for 6, 12, 24, 36, or 48 h to determine the optimal treatment duration. Aβ_1-42_ oligomer preparation is detailed in Supplementary Material.

Neuro-2a (N2a) cells were maintained under the same conditions. Cells were transfected with either an APP-GFP lentiviral vector or a GFP-only lentiviral vector (negative control) following protocols described previously (Xiao et al., 2020). GFP expression was evaluated 3 days post-infection using an inverted fluorescence microscope.

### 2.11 Cell viability and LDH assay

Cell viability was evaluated using the CCK-8 assay. To assess the potential cytotoxicity of ICS II, HT22 cells were seeded in 96-well plates at a density of 5 × 10^3^ cells/well and treated with ICS II alone at concentrations of 0.25, 0.5, 1, 2, 4, 8, or 16 μM for 24 h.

To evaluate its protective effects in the Aβ-induced injury model, HT22 cells were pretreated with ICS II at 0.0625, 0.125, 0.25, 0.5, or 1 μM for 24 h. The ICS II-containing medium was then removed and replaced with fresh medium containing 10 μM Aβ_1-42_ for an additional 24 h.

For the APP-N2a model, GFP fluorescence was examined on day 3 after transfection to confirm transfection efficiency. APP-N2a and control GFP-N2a cells were then reseeded and cultured until they reached 70%–80% confluence, followed by treatment with ICS II at 0.0625, 0.125, 0.25, 0.5, or 1 μM for 24 h.

After the respective treatments, 10 μL of CCK-8 solution was added to each well, and the cells were incubated at 37 °C in the dark for 2 h. Absorbance was measured at 450 nm.

LDH release was measured using the corresponding assay kit to assess the protective effects of ICS II against Aβ-induced injury in HT22 and APP-N2a cells. Absorbance was recorded at 450 nm.

### 2.12 SOD and MDA assays

SOD and malondialdehyde (MDA) levels were quantified using commercial kits. Cells were seeded into 24-well plates at 2 × 10^4^ cells/well, lysed in 1% Triton X-100, subjected to repeated freeze-thaw cycles at −80 °C, and centrifuged. Supernatants were collected, and absorbance was measured at 450 nm (SOD) or 532 nm (MDA).

### 2.13 mtROS and GSH assay

Mitochondrial reactive oxygen species (mtROS) levels were detected using the fluorescent probe MitoSO Red. Cells were divided into five groups: GFP-N2a control (GFP), APP-N2a model (APP), APP + ICS II (ICS II), APP + LY294002 (LY294002), and APP + LY294002 + ICS II (LY294002 + ICS II). For pathway inhibition, APP-N2a cells were pretreated with 10 μM LY294002 for 1 h, followed by co-treatment with or without 0.25 μM ICS II for 24 h to establish the LY294002 and LY294002 + ICS II groups, respectively. After removing the medium, cells were incubated with 5 µM MitoSO Red at 37 °C for 45 min in the dark, washed twice with PBS, and observed under a fluorescence microscope (Zhang L. et al., 2024; Zhou et al., 2021). Fluorescence intensity was measured with ImageJ and normalized to the average intensity of the control group.

GSH levels in cell lysates from the same treatment groups were measured using the corresponding assay kit according to the manufacturer’s instructions.

### 2.14 Apoptosis assay

Apoptosis was evaluated by TUNEL staining (Yang et al., 2024) and Hoechst 33258 staining (Huang et al., 2022). For TUNEL staining, cells were fixed with 4% paraformaldehyde at 4 °C for 25 min, permeabilized with 0.2% Triton X-100 at room temperature for 5 min, and then incubated with 100 μL of TUNEL reaction mixture at 37 °C for 60 min in the dark according to the manufacturer’s instructions, followed by nuclear counterstaining with DAPI. Stained images were captured using a fluorescence microscope to calculate the percentage of TUNEL-positive cells (Cai et al., 2022). For Hoechst 33258 staining, cells were fixed with 4% paraformaldehyde and then stained with 100 μL of Hoechst 33258 for 5 min, and images were acquired under a fluorescence microscope.

### 2.15 JC-1 staining

ΔΨm was assessed using JC-1 fluorescent probe. Cells from five groups (GFP, APP, ICS II, LY294002, and LY294002 + ICS II) were incubated with JC-1 at 37 °C for 20 min in the dark, washed twice with staining buffer, and imaged under a fluorescence microscope.

### 2.16 Morphological analysis of mitochondria

Mitochondrial morphology was examined using an automated multimodal live-cell imaging system (ZIRCON_C600; Zircon Optoelectronics, Suzhou, China). Cells were seeded in 35-mm glass-bottom confocal dishes (BS-20-GJM) at a density of 5 × 10^4^ cells/mL, with 1 mL of cell suspension per dish, and cultured to approximately 50% confluence. The cells were divided into the GFP, APP, ICS II, LY294002, and LY294002 + ICS II groups. After the indicated treatments, the cells were cultured for an additional 24 h before image acquisition under identical imaging conditions.

Mitochondrial morphology was quantitatively analyzed using CellProfiler software (version 4.2.8). Briefly, the acquired images were converted to grayscale using the ColorToGray module, and mitochondrial objects were identified using the IdentifyPrimaryObjects module with consistent thresholding and segmentation parameters across all groups. Mitochondrial object number and major axis length were extracted using the MeasureObjectSizeShape module. Fragmented mitochondria were identified according to a predefined major-axis-length threshold, and the mitochondrial fragmentation percentage was calculated as the number of fragmented mitochondrial objects divided by the total number of mitochondrial objects. All segmented images were visually inspected to exclude nuclei, lipid droplets, cell boundaries, and other obvious non-mitochondrial structures.

### 2.17 Western blot analysis

Cells were washed with ice-cold PBS, lysed, and protein concentrations were determined using the BCA method. Equal amounts of protein were separated by SDS-PAGE, transferred to PVDF membranes, blocked with 5% non-fat milk for 2 h, and incubated overnight at 4 °C with primary antibodies against the following proteins: Bcl-2 (1:500); Bax (1:1000); Caspase-3/cleaved Caspase-3 (1:500); Mfn2 (1:500); Drp1 (1:1000); p-Drp1 (1:1000); Cytc (1:1000); and β-actin (1:5000). Membranes were then incubated with HRP-conjugated secondary antibodies (1:1000) for 2 h at room temperature. Protein bands were visualized using an imaging system, and relative intensities were quantified with ImageJ.

### 2.18 Statistical analysis

Statistical analyses were performed using GraphPad Prism 10.1.2. Data normality was assessed using the Shapiro-Wilk test, and homogeneity of variances was evaluated using the Brown-Forsythe test. For datasets meeting both normality (*P* > 0.05) and homogeneity of variance (*P* > 0.05) assumptions, multiple group comparisons were performed using one-way ANOVA followed by Tukey’s *post hoc* test. When variances were unequal (*P* < 0.05) despite normality, Welch ANOVA was applied. For datasets violating the normality assumption (*P* < 0.05), the Kruskal–Wallis H test was used for overall comparisons, followed by Dunn’s *post hoc* test with Bonferroni correction. Morris water maze escape latency was analyzed using two-way repeated-measures ANOVA followed by Bonferroni’s multiple-comparison test. A value of P < 0.05 was considered statistically significant.

## 3 Results

### 3.1 ICS II improves cognitive performance in APP/PS1 mice

The *in vivo* efficacy of ICS II was evaluated using the MWM. Representative swim paths from the navigation trial are shown in Figure 1B. Compared with the Control group, APP/PS1 mice in the Model group showed persistently prolonged escape latency, indicating impaired spatial learning. In contrast, ICS II-treated mice exhibited significantly shorter escape latencies from day 4 onward (Figure 1C, ***P < 0.001 on day 4 and **P < 0.01 on day 5 vs. Model, F (12, 180) = 1.878). In the probe trial, the Model group showed a longer swimming distance to the former platform location (Figure 1D, ***P < 0.001, vs. Control, F (3, 36) = 8.761), fewer platform crossings, and less time spent in the target quadrant (Figure 1E, ***P < 0.001, F (3, 36) = 6.996, Figure 1F, **P < 0.01, F (3, 36) = 6.032), consistent with marked memory impairment. ICS II treatment significantly improved all of these parameters. No significant differences in swimming speed were observed among groups (Figure 1G, F (3, 36) = 1.972, P = 0.1356), indicating that the behavioral changes were not attributable to altered locomotor activity. Together, these findings show that ICS II improves spatial learning and memory in APP/PS1 mice.

### 3.2 ICS II alleviates histopathological damage in APP/PS1 mice

H&E and Nissl staining were performed to evaluate neuropathological changes in the hippocampus and cortex. In the Model group, H&E staining revealed marked neuronal loss, nuclear shrinkage, and irregular cellular morphology (Figure 1H). Consistently, Nissl staining showed significant reductions in Nissl-positive neurons in the cortex (Figures 1I,J, ***P < 0.001, vs. Control, F (2, 6) = 31.86), hippocampal CA3 region (Figures 1I,K, ***P < 0.001, vs. Control, F (2, 6) = 55.08), and DG (Figures 1I,L, **P < 0.01, vs. Control, F (2, 6) = 16.39). ICS II treatment increased neuronal density, alleviated nuclear shrinkage, improved cellular morphology, and significantly restored Nissl-positive neurons in both the hippocampus and cortex. These results indicate that ICS II attenuates histopathological damage in APP/PS1 mice.

### 3.3 ICS II reduces Aβ deposition in APP/PS1 mice

To determine whether ICS II could reduce Aβ plaque burden in the brains of APP/PS1 mice, immunofluorescence staining was performed to detect Aβ peptide signals in the cortex and hippocampal CA1, CA3, and DG subregions of mice in each group (Figure 1M). The results showed that compared with the Control group, the Model group exhibited abundant and dense Aβ fluorescent signals in the cortex and all hippocampal subregions (Figures 1M–Q, cortex: *P < 0.05, F (2, 6) = 8.565; CA1: **P < 0.01, F (2, 6) = 11.34; CA3: **P < 0.01, F (2, 6) = 11.73; DG: *P < 0.05, F (2, 6) = 8.059). Following ICS II treatment, the distribution range of Aβ-positive plaques was markedly reduced and the fluorescence signals decreased in the aforementioned brain regions (Figures 1M–Q, cortex: *P < 0.05, F (2, 6) = 8.565; CA1: *P < 0.05, F (2, 6) = 11.34; CA3: *P < 0.05, F (2, 6) = 11.73; DG: *P < 0.05, F (2, 6) = 8.059). These findings indicate that ICS II effectively reduces Aβ deposition in multiple subregions of the cortex and hippocampus in APP/PS1 mice and ameliorates cerebral amyloid plaque pathology.

### 3.4 ICS II suppresses neuronal apoptosis in the cortex and hippocampus of APP/PS1 mice

Neuronal apoptosis was assessed by TUNEL staining and cleaved Caspase-3/NeuN double immunofluorescence. TUNEL staining showed that, compared with the Control group, the Model group exhibited significantly increased numbers of TUNEL-positive cells in the cerebral cortex and hippocampal CA1, CA3, and DG regions (Figures 2A–E, cortex: ***P < 0.001, F (2, 6) = 38.38; CA1: ***P < 0.001, F (2, 6) = 34.56; CA3: ***P < 0.001, F (2, 6) = 29.01; DG: ***P < 0.001, F (2, 6) = 52.33), indicating enhanced neuronal apoptosis. ICS II treatment markedly reduced the number of TUNEL-positive cells in these regions (Figures 2A–E, cortex: ***P < 0.001, F (2, 6) = 38.38; CA1: *P < 0.05, F (2, 6) = 34.56; CA3: *P < 0.05, F (2, 6) = 29.01; DG: **P < 0.01, F (2, 6) = 52.33).

**FIGURE 2:**
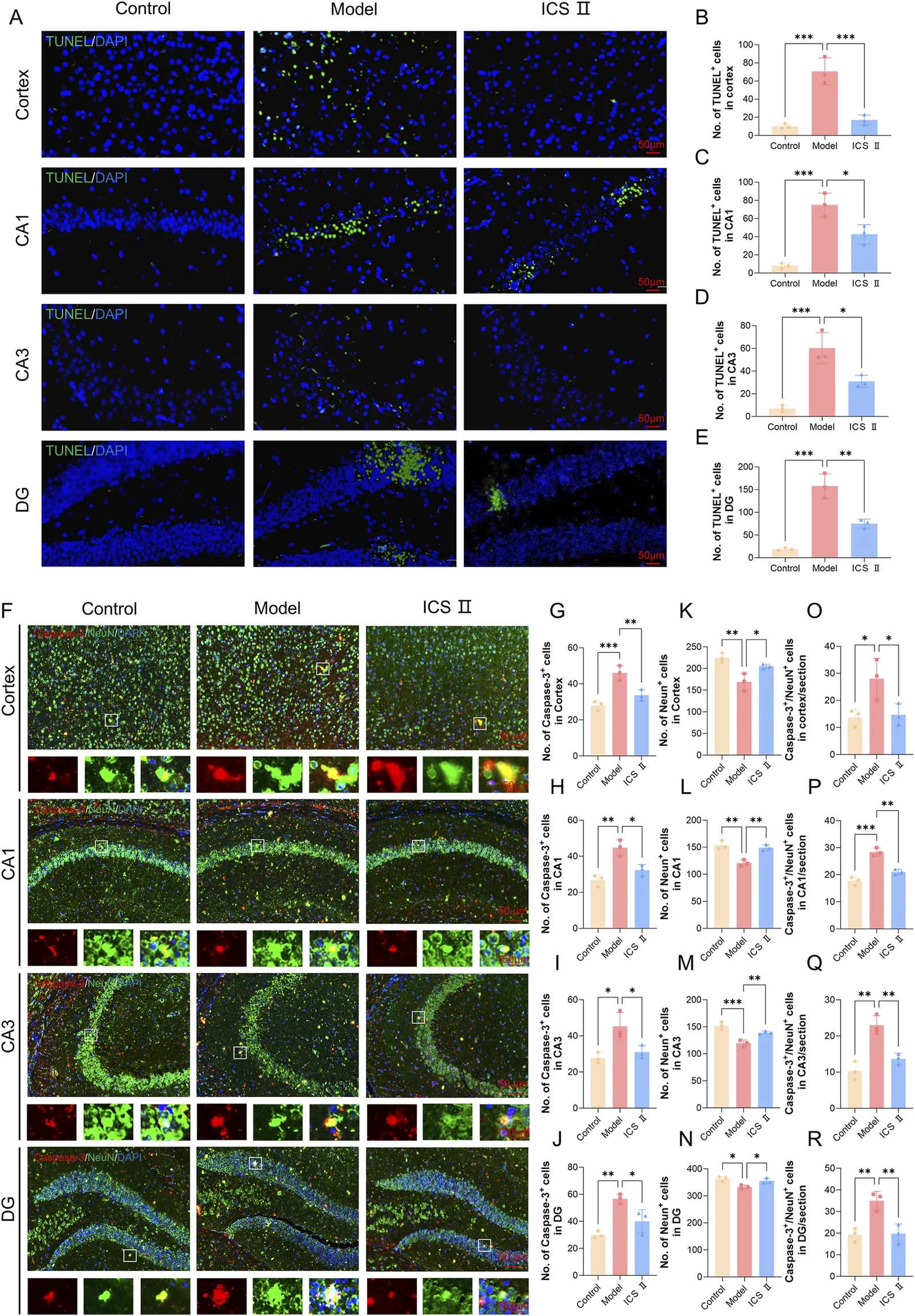
ICS II suppresses neuronal apoptosis in the cortex and Hippocampus of APP/PS1 mice. **(A)** TUNEL staining images in the cerebral cortex and hippocampus, with a scale bar of 50 μm. **(B–E)** Quantification of TUNEL^+^ cells in the cerebral cortex **(B)** hippocampal CA1 **(C)** CA3 **(D)** and DG **(E)** regions. **(F)** Representative images of Caspase-3 and NeuN immunofluorescence in the cerebral cortex and hippocampal regions. Boxed areas indicate Caspase-3^+^/NeuN^+^ cells. Scale bars = 50 μm (original images), 150 μm (magnified views). **(G–J)** Quantification of Caspase-3^+^ cells in the cerebral cortex **(G)** and hippocampal CA1 **(H)** CA3 **(I)** and DG **(J)** regions. **(K–N)** Quantification of NeuN^+^ cells in the cerebral cortex **(K)** and hippocampal CA1 **(L)** CA3 **(M)** and DG **(N)** regions. **(O–R)** Quantification of Caspase-3^+^/NeuN^+^ cells in the cerebral cortex **(O)** and hippocampal CA1 **(P)** CA3 **(Q)** and DG **(R)** regions. Statistical significance: *P < 0.05, **P < 0.01, ***P < 0.001. Data are presented as mean ± SD (n = 3 mice/group).

These findings were further supported by cleaved Caspase-3/NeuN double immunofluorescence. Relative to the Control group, the Model group displayed increased cleaved Caspase-3-positive cells (Figures 2F–J, cortex: ***P < 0.001, F (2, 6) = 23.58; CA1: **P < 0.01, F (2, 6) = 19.06; CA3: *P < 0.05, F (2, 6) = 9.954; DG: **P < 0.01, F (2, 6) = 17.13), decreased NeuN-positive cells (Figures 2F,K–N, cortex: **P < 0.01, F (2, 6) = 13.45; CA1: **P < 0.01, F (2, 6) = 20.28; CA3: ***P < 0.001, F (2, 6) = 25.81; DG: *P < 0.05, F (2, 6) = 8.856), and increased cleaved Caspase-3/NeuN double-positive cells (Figures 2F,O–R, cortex: *P < 0.05, F (2, 6) = 6.888; CA1: ***P < 0.001, F (2, 6) = 47.29; CA3: **P < 0.01, F (2, 6) = 24.77; DG: **P < 0.01, F (2, 6) = 14.82) in both the cortex and hippocampus, consistent with enhanced neuronal injury and apoptosis. ICS II treatment significantly reduced cleaved Caspase-3-positive cells, increased NeuN-positive cells, and decreased double-positive cells across these brain regions (Figures 2F–R). Collectively, these data indicate that ICS II suppresses neuronal apoptosis and preserves neuronal survival in APP/PS1 mice.

### 3.5 RNA-sequencing analysis identifies PI3K/AKT signaling as a candidate pathway associated with ICS II treatment

To explore the molecular basis of ICS II action *in vivo*, RNA-sequencing analysis was performed. A total of 373 differentially expressed genes (DEGs) were identified between the Model and Control groups (215 upregulated and 158 downregulated), whereas 283 DEGs were identified between the ICS II and Model groups (49 upregulated and 234 downregulated) (Figures 3A,B; Supplementary File 4). To investigate the reversal effect of ICS II on dysregulated gene expression in AD model mice, we focused on 67 DEGs that were abnormally expressed in the model group but reversed after ICS II treatment, and performed GO and KEGG functional enrichment analyses on these genes. GO enrichment analysis revealed that these reversed genes were primarily involved in biological processes (BP) such as regulation of neuronal death, macrophage differentiation and migration, and inflammatory responses. Cellular component (CC) annotation indicated significant enrichment in membrane microdomains and lysosomal lumen, while molecular function (MF) analysis showed enrichment in cysteine-type peptidase activity and growth factor binding (Figure 3C; Supplementary File 5). KEGG pathway enrichment analysis demonstrated that the reversed genes were significantly enriched in apoptosis, PI3K-AKT, MAPK, and lysosome signaling pathways (Figure 3D; Supplementary File 6).

**FIGURE 3:**
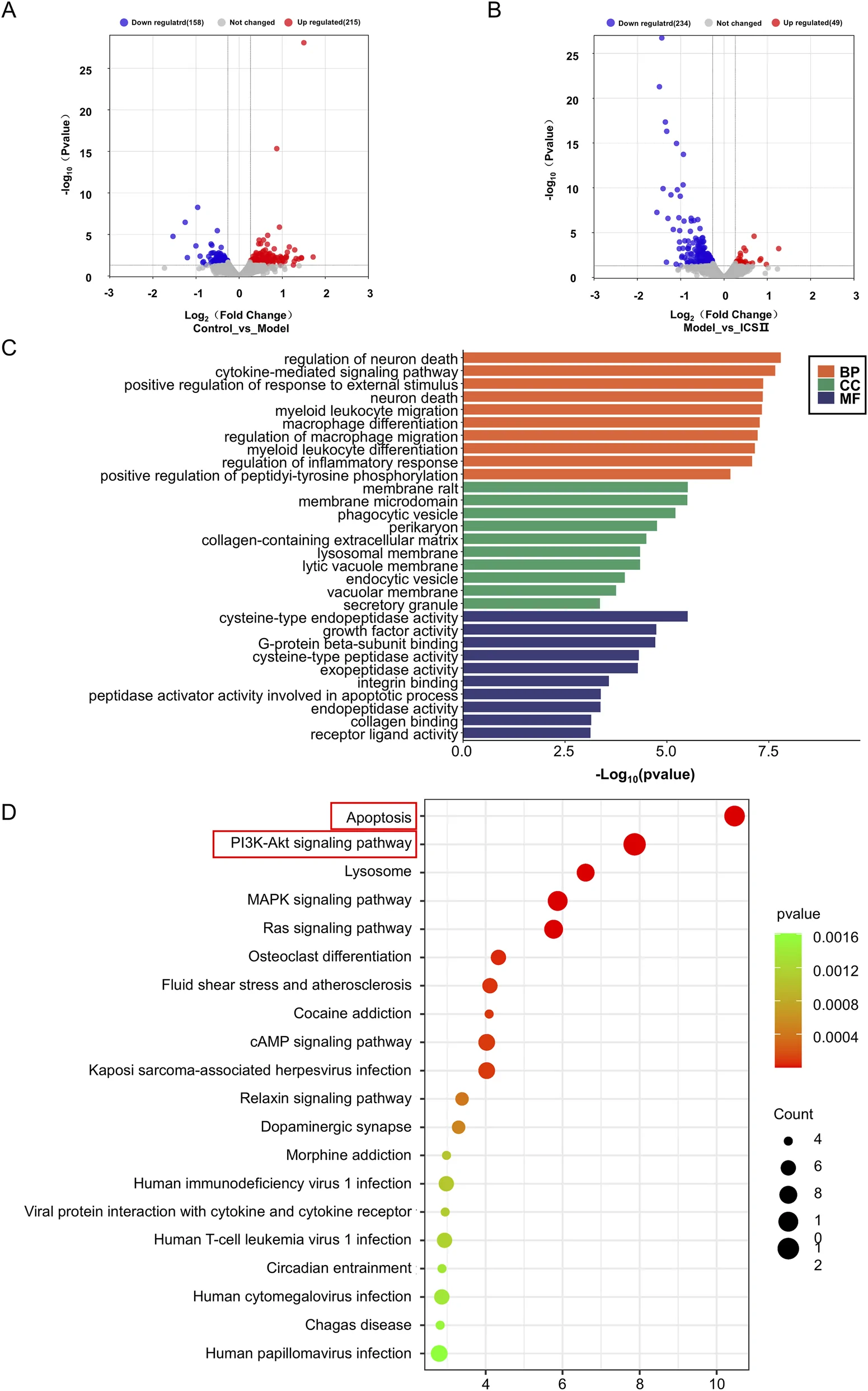
RNA-sequencing analysis of the Hippocampus after ICS II treatment. **(A)** Volcano plot of Control group vs. Model group. **(B)** Volcano plot of Model group vs. ICS II group. **(C)** GO enrichment analysis of the 67 reversed genes. **(D)** KEGG enrichment analysis of the 67 reversed genes. n = 3 mice/group.

### 3.6 ICS II protects Aβ-injured HT22 cells

To exclude the possibility that the observed protective effects were attributable to intrinsic cytotoxicity of ICS II, we first evaluated the effect of ICS II alone on normal HT22 cells. As shown in Supplementary Figure 1, treatment with ICS II at concentrations ranging from 0.25 to 16 μM for 24 h did not significantly affect cell viability in normal HT22 cells, indicating that ICS II alone did not exert apparent cytotoxicity within the tested concentration range.

Subsequently, the CCK-8 assay was employed to determine the optimal conditions for Aβ_1-42_-induced injury and ICS II intervention in HT22 cells. Treatment with 10 μM Aβ_1-42_ for 24 h reduced HT22 cell viability to approximately 60% (Figure 4A, **P < 0.01, vs. Control, F (5, 30) = 9.606). Exposure to 10 μM Aβ_1-42_ for 24, 36, or 48 h significantly decreased cell viability (Figure 4B), and 24 h was selected for subsequent experiments because it produced moderate and reproducible injury. ICS II at 0.25 μM showed the strongest protective effect under these conditions (Figure 4C, *P < 0.05, vs. Model, F (6, 35) = 16.34). Based on these results, 0.125, 0.25, and 0.5 μM were selected as the low, medium, and high concentrations, respectively, for subsequent protective experiments.

**FIGURE 4:**
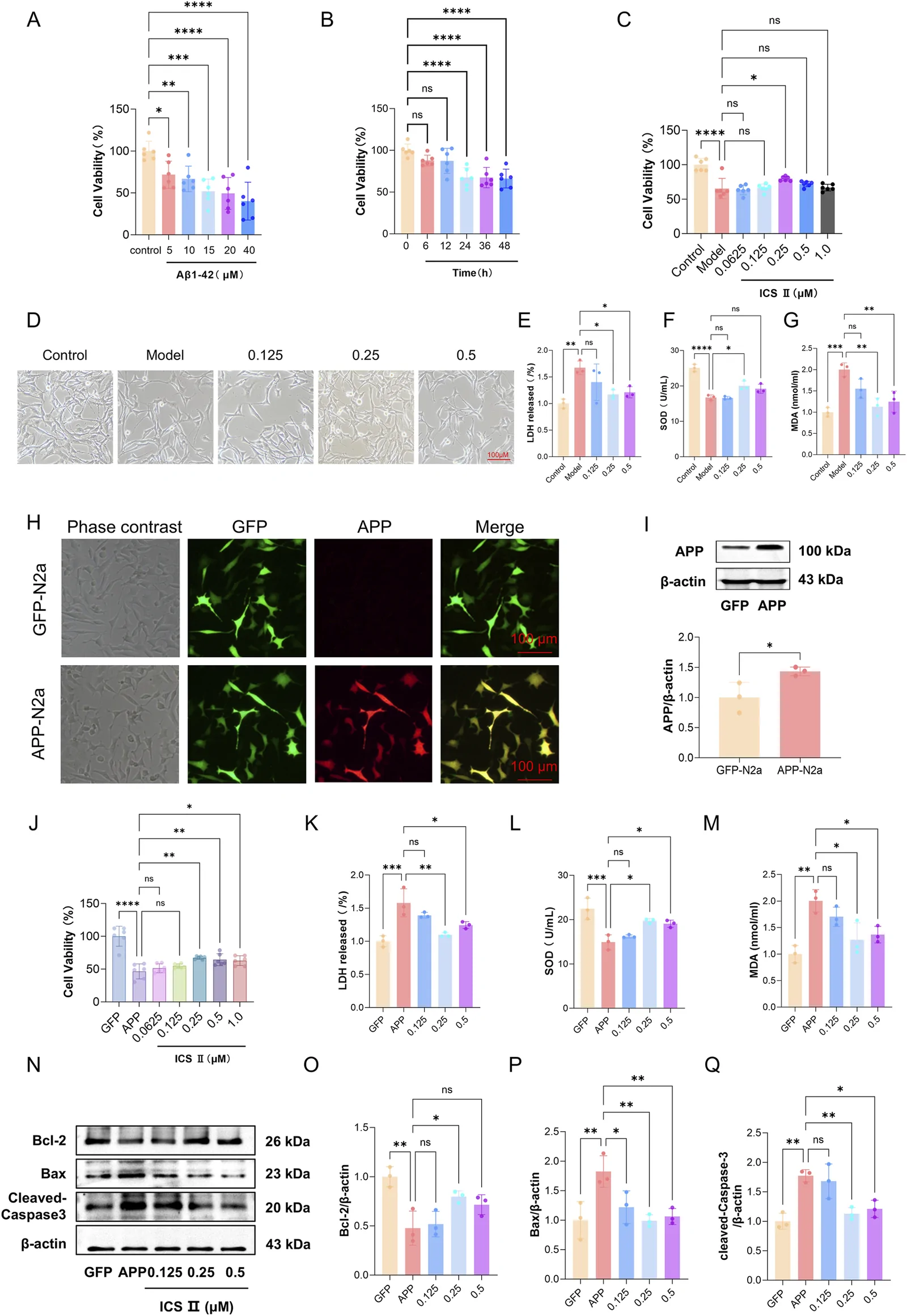
Protective effect of ICS II on Aβ-HT22 and APP-N2a Cells. **(A)** Cell viability assessed by CCK-8 assay after HT22 cells were incubated with varying concentrations (0, 5, 10, 15, 20, 40 μM) of Aβ_1-42_ for 24 h, to determine the optimal modeling concentration (n = 6). **(B)** Cell viability assessed by CCK-8 assay after HT22 cells were co-incubated with 10 μM Aβ_1-42_ for different durations (0, 6, 12, 24, 36, 48 h), to determine the optimal modeling duration (n = 6). **(C)** Cell viability assessed by CCK-8 assay after the successful establishment of the Aβ-HT22 cell model, followed by treatment with different concentrations of ICS II (0.0625, 0.125, 0.25, 0.5, 1 μM) (n = 6). **(D)** The effect of ICS II on the morphology of Aβ-HT22 cells (n = 3). **(E)** Measurement of LDH release (n = 3). **(F)** Statistical graph of SOD activity (n = 3). **(G)** Statistical graph of MDA content (n = 3). **(H)** Transfection of N2a cells with GFP and APP595/596 plasmids (n = 3). **(I)** Western blot analysis of APP protein levels in each group (n = 3). **(J,K)** Cell viability detected by CCK-8 and LDH assays (n = 6). **(L)** Statistical graph of SOD activity (n = 3). **(M)** Statistical graph of MDA content (n = 3). **(N)** Apoptosis detected by Western blot. **(O)** Relative protein expression of Bcl-2 (n = 3). **(P)** Relative protein expression of Bax (n = 3). **(Q)** Relative protein expression of cleaved-Caspase 3 (n = 3). Statistical significance: *P < 0.05; **P < 0.01; ***P < 0.001; ****P < 0.0001; ns: no significant difference. Data are presented as mean ± SD.

On this basis, after Aβ_1-42_ induction, HT22 cell viability was significantly decreased, LDH release was markedly increased (Figure 4E, **P < 0.01, vs. Control, F (4, 10) = 6.154), and cell morphology changed from normal spindle-like to spherical and shrunken (Figure 4D), indicating that Aβ_1-42_ successfully induced neuronal injury. Meanwhile, Aβ_1-42_ induced oxidative stress, manifested as significantly decreased SOD activity (Figure 4F, ****P < 0.0001, vs. Control, F (4, 10) = 33.88) and increased MDA content (Figure 4G, ***P < 0.001, vs. Control, F (4, 10) = 12.74). ICS II pretreatment reversed the above damage. Specifically, ICSII significantly raised cell viability, reduced LDH release, improved cell morphology, and markedly alleviated oxidative stress, as reflected by increased SOD activity and decreased MDA content, with the most pronounced improvement observed in the medium-concentration (0.25 μM) group. Collectively, these results demonstrate that ICSII effectively attenuates Aβ_1-42_-induced injury in HT22 cells.

### 3.7 ICS II protects APP-overexpressing N2a cells

The APP-N2a cell model was confirmed by fluorescence microscopy and Western blotting (Figures 4H,I). CCK-8 analysis identified 0.25 μM as the optimal concentration of ICS II (Figure 4J, **P < 0.01, vs. APP, F (6, 35) = 23.26). Based on these results, 0.125, 0.25, and 0.5 μM were selected as the low, medium, and high concentrations, respectively, for subsequent mechanistic experiments.

Under these conditions, compared with the APP group, ICS II treatment significantly reduced LDH release (Figure 4K, **P < 0.01, vs. APP, F (4, 10) = 13.91), elevated SOD activity (Figure 4L, *P < 0.05, vs. APP, F (4, 10) = 13.77), and decreased MDA content (Figure 4M, *P < 0.05, vs. APP, F (4, 10) = 9.848) in APP-N2a cells. Furthermore, ICSII significantly downregulated the expression of the pro-apoptotic proteins Bax (Figures 4N,P, **P < 0.01, vs. APP, F (4, 10) = 6.705) and cleaved Caspase-3 (Figures 4N,Q, **P < 0.01, vs. APP, F (4, 10) = 12.18), while upregulating the expression of the anti-apoptotic protein Bcl-2 (Figures 4N,O, *P < 0.05, vs. APP, F (4, 10) = 9.660). Collectively, these results demonstrate that ICSII protects APP-N2a cells by attenuating oxidative stress and suppressing apoptosis, with 0.25 μM being the optimal concentration.

### 3.8 PI3K/AKT inhibition attenuates the cytoprotective and anti-apoptotic effect of ICS II in APP-N2a cells

To determine whether PI3K/AKT signaling contributes to the pro-survival effects of ICS II, APP-N2a cells were treated with the PI3K inhibitor LY294002 (Huang et al., 2021; Liu et al., 2024). CCK-8 assay showed that ICS II significantly increased cell viability compared with the APP group (Figure 5C, **P < 0.01, F (4, 25) = 9.714), whereas LY294002 largely abolished this pro-survival effect (Figure 5C, *P < 0.05, LY294002+ ICS II vs. ICS II, F (4, 25) = 9.714).

**FIGURE 5:**
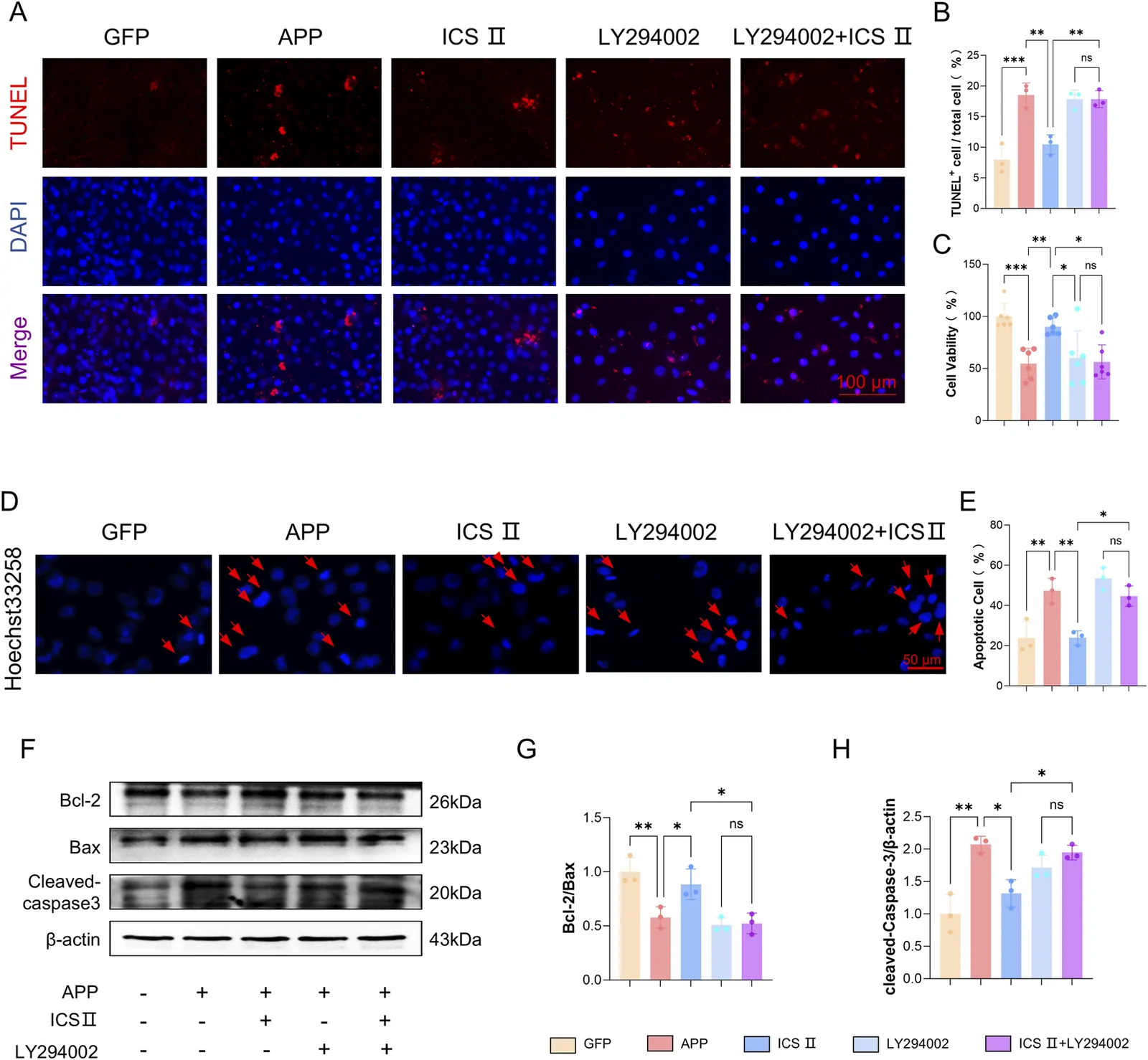
PI3K/AKT inhibition attenuates the cytoprotective and anti-apoptotic effects of ICS II in APP-N2a cells. **(A)** Apoptosis cells determined with TUNEL staining (red). (scale bar = 50 μm). **(B)** Statistical results of TUNEL positive neurons/total neurons. (n = 3). **(C)** Cell viability measured by CCK-8 assay (n = 6). **(D)** Representative images of Hoechst 33258 staining (scale bar = 50 μm). **(E)** Quantitative analysis of Hoechst 33258-positive cells (n = 3). **(F–H)** Western blot analysis of Bcl-2, Bax, and cleaved-caspase 3 protein levels in each group (n = 3). Statistical significance: *P < 0.05; **P < 0.01; ***P < 0.001; ns: no significant difference. Data are presented as mean ± SD.

Apoptosis was further evaluated by TUNEL staining, Hoechst 33258 staining, and Western blotting. Compared with the APP group, ICS II significantly reduced the proportions of TUNEL-positive cells (Figures 5A,B, **P < 0.01, F (4, 10) = 22.43) and Hoechst 33258-positive cells (Figures 5D,E, **P < 0.01, F (4, 10) = 16.27), indicating attenuation of apoptotic injury. LY294002 markedly weakened the anti-apoptotic effects of ICS II. The ICS II + LY294002 group showed higher proportions of TUNEL-positive (Figures 5A,B, **P < 0.01, F (4, 10) = 22.43) and Hoechst 33258-positive cells (Figures 5D,E, *P < 0.05, F (4, 10) = 16.27) than the ICS II group, while no significant differences were observed between the LY294002 and ICS II + LY294002 groups for these apoptosis-related endpoints. Consistently, Western blotting showed that ICS II increased the Bcl-2/Bax ratio and reduced cleaved-caspase 3 expression, whereas LY294002 attenuated these effects (Figures 5F,G, *P < 0.05, F (4, 10) = 12.88; Figures 5F,H, *P < 0.05, F (4, 10) = 11.76). In addition, the LY294002 and ICS II + LY294002 groups showed similar changes in apoptosis-related proteins, suggesting that inhibition of PI3K/AKT signaling limited the protective effect of ICS II. Together, these findings indicate that the anti-apoptotic effect of ICS II is functionally associated with, and at least partly dependent on, PI3K/AKT pathway activation.

### 3.9 PI3K/AKT inhibition attenuates the protective effect of ICS II on mitochondrial function and oxidative stress

Given the close link between mitochondrial dysfunction and apoptosis, we next assessed the effects of ICS II on mitochondrial function and oxidative stress in APP-N2a cells. ICS II significantly reduced mtROS levels (Figures 6A,B, ***P < 0.001, vs. APP, F (4, 10) = 140.1) and increased GSH content (Figure 6C, *P < 0.05, vs. APP, F (4, 15) = 22.26). JC-1 staining showed a marked decrease in red/green fluorescence ratio in APP cells, indicating loss of ΔΨm, whereas ICS II restored ΔΨm (Figures 6D–G). This effect was markedly attenuated by LY294002 (Figure 6G, **P < 0.01, vs. ICS II, F (4, 10) = 28.76).

**FIGURE 6:**
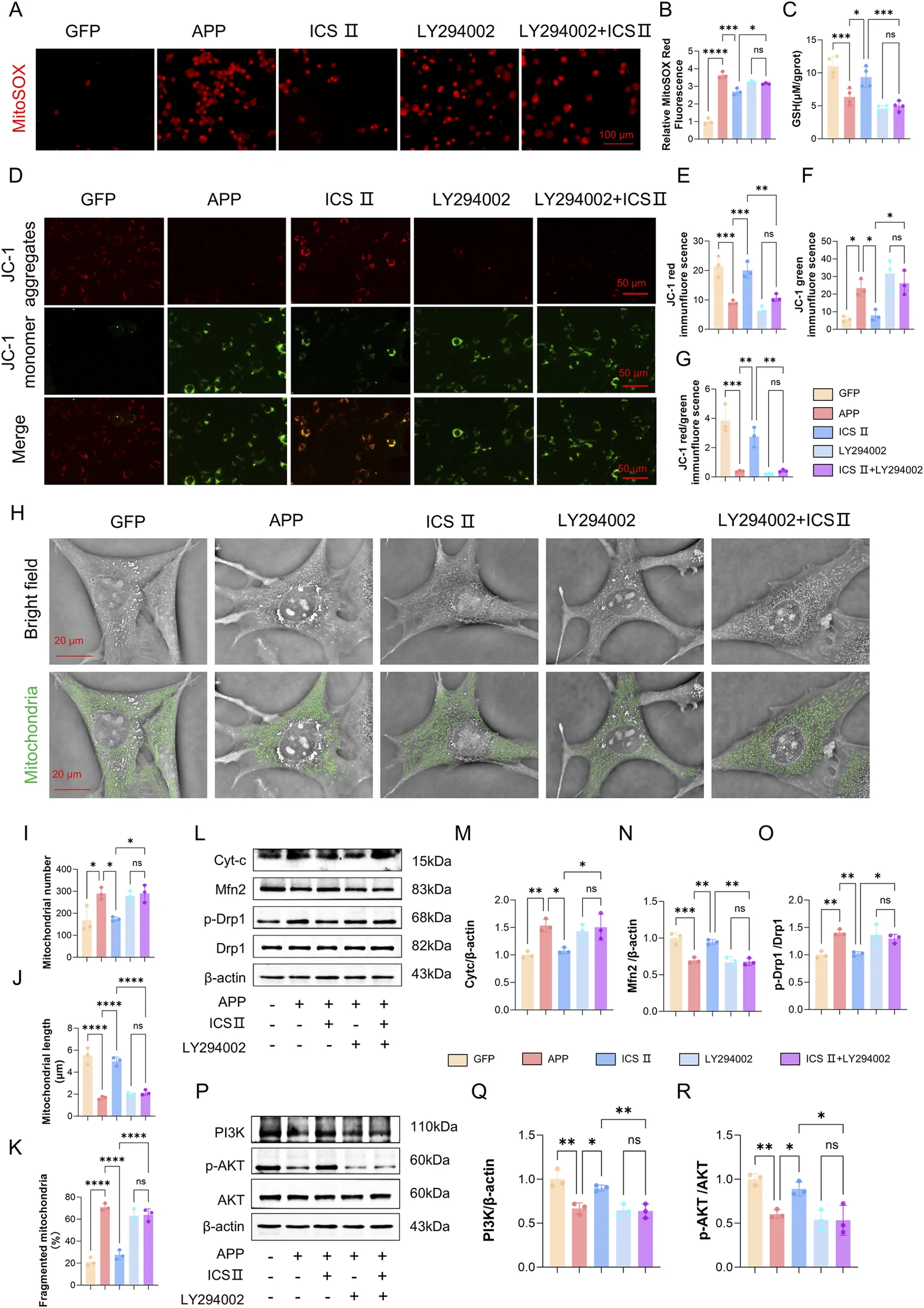
PI3K/AKT inhibition attenuates the protective effects of ICS II on mitochondrial function and oxidative stress in APP-N2a cells. **(A)** Representative images of MitoSox-Red (red color) (scale bar = 50 μm). **(B)** Relative fluorescence intensity of MitoSox-Red (n = 3). **(C)** Statistical graph of GSH levels (n = 4). **(D)** Representative images of JC-1 aggregates, JC-1 monomers, and their merge (scale bar = 50 μm). **(E)** Quantitative analysis of JC-1 aggregate fluorescence intensity (n = 3). **(F)** Quantitative analysis of JC-1 monomer fluorescence intensity (n = 3). **(G)** Quantitative analysis of the JC-1 aggregate/monomer fluorescence intensity ratio (n = 3). **(H)** Representative images of mitochondrial morphology (scale bar = 20 μm). **(I)** Quantification of mitochondrial number (n = 3). **(J)** Quantification of mitochondrial length (n = 3). **(K)** Percentage of fragmented mitochondria (n = 3). **(L–O)** Western blot analysis of Cytochrome c (Cyt c), Mitofusin-2 (Mfn2), and phosphorylated Drp1 (p-Drp1) protein levels in each group (n = 3). **(P–R)** Western blot analysis of PI3K, p-AKT, and AKT protein levels in each group (n = 3). Statistical significance: *P < 0.05; **P < 0.01; ***P < 0.001; ****P < 0.0001; ns: no significant difference. Data are presented as mean ± SD.

Consistent with these functional changes, we next examined mitochondrial morphology. Compared with the GFP group, the APP group showed pronounced mitochondrial fragmentation (Figure 6H), as indicated by increased punctate and short rod-like mitochondria, increased mitochondrial object number (Figure 6I, *P < 0.05, F (4,10) = 9.440), reduced mean mitochondrial length (Figure 6J, ****P < 0.0001, F (4,10) = 62.31), and elevated mitochondrial fragmentation (Figure 6K, ****P < 0.0001, F (4,10) = 73.50). ICS II treatment markedly improved mitochondrial morphology, with increased tubular mitochondria, reduced mitochondrial object number and fragmentation (Figure 6I, *P < 0.05, F (4,10) = 9.440; Figure 6K, ****P < 0.0001, F (4,10) = 73.50), and increased mean mitochondrial length (Figure 6J, ****P < 0.0001, F (4,10) = 62.31). However, the PI3K/AKT inhibitor LY294002 attenuated the protective effect of ICS II, leading to increased mitochondrial fragmentation.

Cyt c levels were increased in APP group relative to GFP group and were reduced by ICS II treatment; this reduction was weakened by LY294002 (Figures 6L,M, *P < 0.05, vs. ICS II, F (4, 10) = 10.07). In parallel, APP group exhibited decreased Mfn2 expression (Figures 6L,N, ***P < 0.001, vs. GFP, F (4, 10) = 22.84) and increased p-Drp1 expression (Figures 6L,O, **P < 0.01, vs. GFP, F (4, 10) = 11.46), consistent with disrupted mitochondrial dynamics. ICS II reversed these changes by upregulating Mfn2 and downregulating p-Drp1, whereas LY294002 attenuated these effects (Figures 6L,N, **P < 0.01, vs. ICS II, F (4, 10) = 22.84; Figures 6L,O, *P < 0.05, vs. ICS II, F (4, 10) = 11.46). Taken together, these results indicate that ICS II preserves mitochondrial homeostasis, at least in part, in a PI3K/AKT-dependent manner.

### 3.10 ICS II activates PI3K/AKT signaling in APP-N2a cells

To further assess the involvement of PI3K/AKT signaling, the expression of pathway-related proteins was examined by Western blotting. Compared with GFP group, APP group showed reduced PI3K expression and a lower p-AKT/AKT ratio (Figures 6P–R). ICS II treatment significantly increased PI3K expression and AKT phosphorylation (Figures 6P,Q, *P < 0.05, vs. APP, F (4, 10) = 15.59; Figures 6P,R, *P < 0.05, vs. APP, F (4, 10) = 13.10), whereas LY294002 reversed these effects (Figures 6P,Q, **P < 0.01, vs. ICS II, F (4, 10) = 15.59; Figures 6P,R, *P < 0.05, vs. ICS II, F (4, 10) = 13.10). Total AKT levels remained unchanged (*P* > 0.05). These findings support the conclusion that activation of PI3K/AKT signaling contributes to the protective effects of ICS II in APP-N2a cells.

## 4 Discussion

In the present study, we investigated whether ICS II confers neuroprotection against AD-related neuronal injury and explored the underlying mechanisms. Our results demonstrate that ICS II not only ameliorates cognitive deficits and histopathological damage in APP/PS1 mice, but also attenuates Aβ burden and reduces neuronal apoptosis in the hippocampus and cortex. In complementary cellular models, ICS II preserved mitochondrial morphology and function, suppressed oxidative stress, and inhibited intrinsic apoptotic signaling. Moreover, RNA-sequencing profiling combined with pharmacological inhibition using LY294002 identified the PI3K/AKT pathway as a critical functional mediator of these mitochondrial protective effects. Collectively, these findings establish that ICS II protects against AD-related neurodegeneration, at least in part, through PI3K/AKT-dependent regulation of mitochondrial dynamics and apoptosis (Figure 7).

**FIGURE 7:**
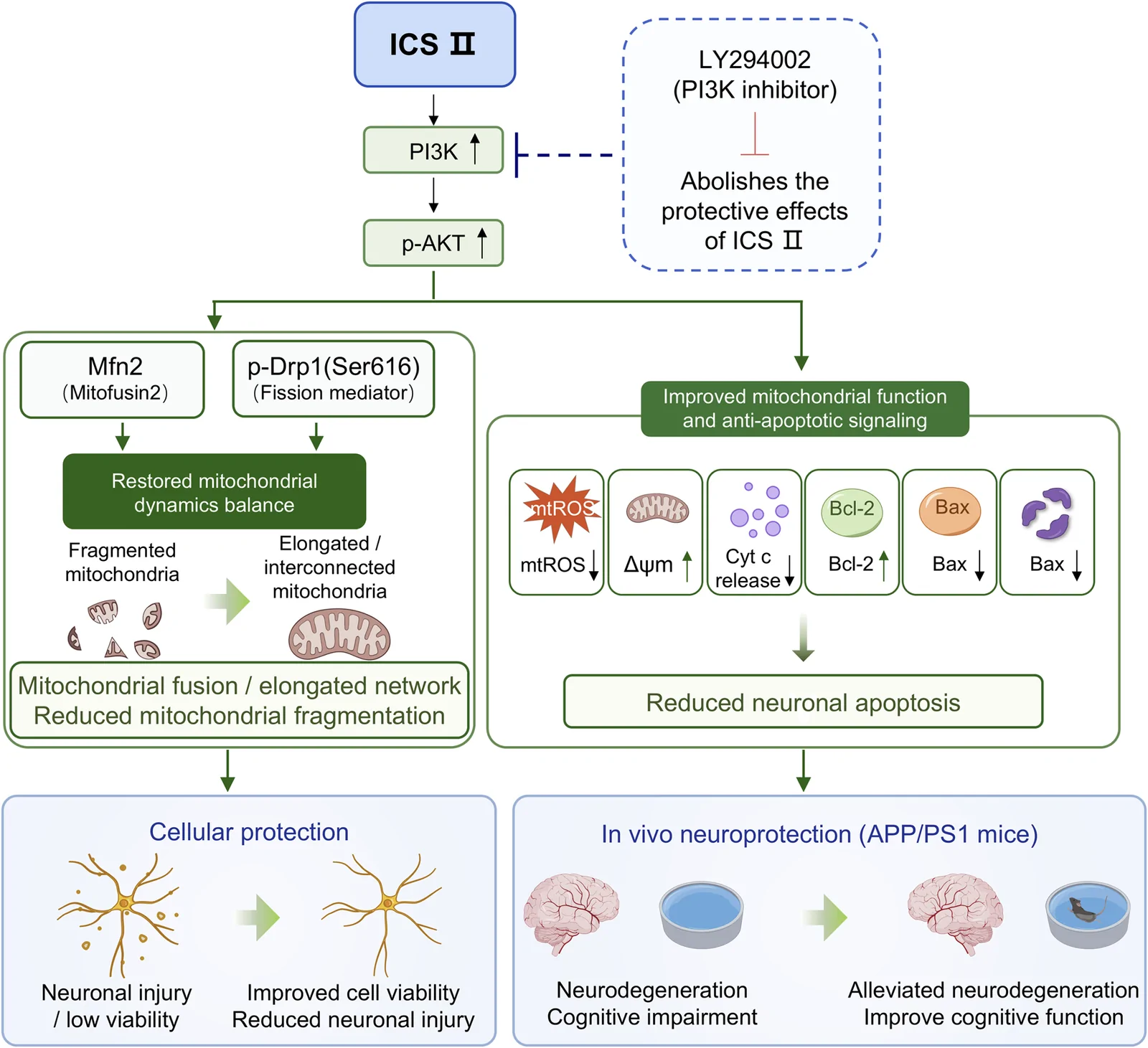
Proposed mechanism of ICS II-mediated neuroprotection in AD models. ICS II activates the PI3K/AKT signaling pathway, accompanied by increased Mfn2 expression and reduced Drp1 phosphorylation at Ser616, which favors restoration of mitochondrial dynamics and suppression of excessive mitochondrial fragmentation. ICS II also preserves mitochondrial membrane potential, reduces mitochondrial superoxide accumulation and cytochrome c release, and suppresses mitochondria-associated apoptotic signaling. Pharmacological inhibition of PI3K with LY294002 attenuates these effects, supporting the involvement of PI3K/AKT signaling in ICS II-mediated regulation of mitochondrial homeostasis and neuronal survival. These cellular effects are associated with reduced neuronal injury and improved cognitive performance in AD models.

Previous studies have shown that ICS II exerts neuroprotective actions through multiple mechanisms, including antioxidative, anti-inflammatory, anti-apoptotic, and neurotrophic effects. For example, Liu et al. (Liu et al., 2018) reported that ICS II ameliorates Aβ-induced cognitive deficits through modulation of the BDNF/TrkB/CREB pathway and inhibition of phosphodiesterase-5. Other studies have shown that ICS II attenuates neuronal injury in cerebral ischemia models (Gao et al., 2023), inhibits hippocampal neuronal apoptosis (He et al., 2018), and promotes neurological recovery after brain injury (Liu et al., 2018). In addition, ICS II also improved learning and memory and promoted endogenous neurogenesis in AD-related models (Bai et al., 2025; Xiao et al., 2022; Xiao et al., 2021). Building on these observations, the present study extends the known actions of ICS II beyond previously reported neurotrophic and neurogenic mechanisms by providing evidence that ICS II is also associated with the regulation of mitochondrial dynamics and mitochondria-related apoptosis in AD models. This extension is important because neuronal protection in AD depends not only on trophic support and regenerative capacity, but also on preservation of mitochondrial integrity under sustained pathological stress.

Mitochondrial dysfunction is increasingly recognized as a central contributor to AD pathogenesis (Blagov et al., 2022). In addition to serving as the primary source of ATP, mitochondria regulate redox homeostasis, calcium buffering, and apoptotic signaling, all of which are essential for neuronal survival (Calvo-Rodriguez and Bacskai, 2021). Because neurons are highly energy-dependent and structurally polarized cells, they are particularly vulnerable to mitochondrial damage. Among the multiple dimensions of mitochondrial dysfunction, disruption of mitochondrial dynamics-namely the balance between fusion and fission-has emerged as a particularly important event in AD (Bera et al., 2022; Flannery and Trushina, 2019; Woo et al., 2021). Excessive mitochondrial fission, often accompanied by reduced fusion, promotes mitochondrial fragmentation, loss of ΔΨm, oxidative stress, bioenergetic failure, and ultimately activation of apoptotic pathways (Reddy et al., 2012; Swerdlow et al., 2014; Swerdlow and Khan, 2004). In this context, our data show that ICS II upregulated Mfn2 (Figures 6L,N), decreased Drp1 phosphorylation at Ser616 (Figures 6L,O), improved mitochondrial morphology (increasing tubular mitochondria and reducing fragmentation, Figure 6H), restored ΔΨm (Figures 6D–G), reduced mtROS accumulation (Figures 6A,B), and decreased Cyt c release (Figures 6L,M). This fundings suggest that preservation of mitochondrial homeostasis may represent an important component of its neuroprotective action.

Accumulating evidence indicates that Drp1 expression and activity are frequently elevated in AD patient brain tissue and in experimental models, and that this dysregulation contributes to mitochondrial fragmentation, synaptic dysfunction, impaired energy metabolism, and neuronal loss (Bhatti et al., 2023; Wang et al., 2020). Moreover, Drp1 dysregulation may interact with core AD pathological events, including Aβ accumulation and abnormal Tau modification, thereby further aggravating mitochondrial damage and neurodegeneration (Bhatti et al., 2023). In this context, our findings provide additional support for the view that modulation of mitochondrial dynamics, particularly suppression of pathological Drp1-associated mitochondrial fragmentation, may represent a therapeutically relevant strategy for AD (Wang et al., 2020). At the same time, it should be noted that the present study primarily demonstrates changes in Drp1 phosphorylation at Ser616 under the current experimental conditions; whether ICS II also regulates Drp1 through other phosphorylation sites, subcellular translocation, or other additional post-translational mechanisms requires further investigation.

KEGG pathway analysis revealed that the DEGs reversed by ICS II were significantly enriched in apoptosis and PI3K/AKT signaling pathways (Figure 3D). The PI3K/AKT pathway is a major pro-survival signaling cascade that regulates cell survival, oxidative stress responses, and apoptosis, and has been closely linked to mitochondrial homeostasis (Wang et al., 2025; Xu et al., 2024). Previous studies have shown that PI3K/AKT signaling influences the balance between pro-apoptotic and anti-apoptotic proteins, including Bax and Bcl-2, and thereby modulates caspase-dependent cell death (Li et al., 2018; Zhao et al., 2020). This suggested a potential involvement of the PI3K/AKT-mitochondrial apoptosis axis in the neuroprotective effect of ICS II. Subsequent *in vitro* experiments showed that ICS II increased PI3K expression and AKT phosphorylation. These changes were accompanied by improved cell survival, reduced apoptosis, restoration of mitochondrial morphology and function, and correction of Mfn2/Drp1 imbalance. Importantly, compared with ICS II treatment alone, co-treatment with the PI3K inhibitor LY294002 markedly attenuated these protective effects. Overall, under the present experimental conditions, the cell protection effect of ICS II is closely associated with PI3K/AKT signaling pathway.

At the same time, the interpretation of this pathway should remain appropriately cautious. Although LY294002 effectively weakened the protective actions of ICS II, the present data do not establish that PI3K/AKT is the direct molecular target of ICS II, nor do they demonstrate that AKT directly regulates Drp1 in this context. The current results support a PI3K/AKT-dependent mechanism, but the precise molecular intermediates linking PI3K/AKT activation to mitochondrial dynamics remain to be clarified. For example, whether AKT influences Drp1 primarily through transcriptional regulation, post-translational modification, or indirect control via other mitochondrial regulatory factors warrants further study. This point is particularly important because the PI3K/AKT pathway is known to engage in extensive crosstalk with multiple signaling networks relevant to AD, including mTOR, AMPK, and GSK-3β. Therefore, the pathway identified here should be interpreted as a functionally relevant mechanism rather than a fully defined linear signaling axis.

Another important aspect of the present study is the coupling between mitochondrial preservation and suppression of intrinsic apoptosis. Mitochondrial damage is a major trigger of intrinsic apoptotic signaling, in part through mitochondrial membrane depolarization, oxidative stress, release of Cyt c, and activation of downstream caspases. Consistent with this framework, ICS II reduced mtROS, increased antioxidant capacity, preserved ΔΨm, decreased Cyt c-associated apoptotic signaling, and reduced Caspase-3 expression. These changes were accompanied by an increased Bcl-2/Bax ratio and reduced neuronal apoptosis both *in vitro* and *in vivo*. Thus, rather than acting only at the terminal stage of apoptosis, ICS II appears to influence upstream mitochondrial events that are closely linked to apoptotic execution. This feature may be particularly relevant in AD, where chronic mitochondrial stress and progressive neuronal vulnerability precede overt neuronal loss.

From a translational perspective, the present findings support ICS II as a natural product-derived lead compound for AD-related neurodegeneration. Unlike most currently approved AD drugs, such as donepezil, rivastigmine, galantamine, and memantine, which primarily provide symptomatic benefit without effectively interrupting neurodegenerative progression (Burns et al., 2022; Cui et al., 2019; Hajjo et al., 2022; Marco-Contelles et al., 2006; Matsunaga et al., 2018; Rösler et al., 1999), ICS II showed protective effects at multiple levels in our experimental systems, including behavioral improvement, attenuation of neuropathological damage, preservation of mitochondrial function, and reduction of apoptosis. Although these preclinical findings do not establish disease-modifying efficacy, they do suggest that ICS II may have value as a multitarget neuroprotective compound acting on mitochondrial dysfunction-related mechanisms that are highly relevant to AD pathology. In addition, as a naturally derived bioactive metabolite from *Epimedium*, ICS II may provide a useful pharmacological scaffold for the development of mitochondria-oriented therapeutic strategies. However, we acknowledge that these correlative findings do not definitively establish a causal mediating role of mitochondrial dynamics. Further studies employing direct genetic manipulation (e.g., Mfn2 knockdown or enforced Drp1 activation) are warranted to determine whether restoration of mitochondrial dynamics is indispensable for the neuroprotective effects of ICS II.

Several limitations should be acknowledged. First, the MWM test is inherently stressful, and the anxiolytic/antidepressant-like effects of ICS II may confound performance via thigmotaxis or floating. Although our data suggest minimal confounds, future studies should systematically quantify these behaviors. Second, only male APP/PS1 mice were used in the present study, and the mice were examined at 6–7 months of age, which represents an early-to-moderate disease stage. Given the well-recognized sex differences in AD pathology and behavioral deficits, as well as the potential for age-dependent effects, future studies including female APP/PS1 mice and extending to older ages are needed to determine whether the effects and mechanisms of ICS II are sex- or age-dependent.

## 5 Conclusion

In summary, our findings demonstrate that ICS II exerts protective effects against AD-related neuronal injury in APP/PS1 mice and neuronal cell models. ICS II treatment improved cognitive performance and neuropathological alterations and was associated with activation of the PI3K/AKT signaling pathway, restoration of mitochondrial dynamics, preservation of mitochondrial function, and suppression of mitochondria-associated apoptosis. Pharmacological inhibition of PI3K attenuated these protective effects, supporting the involvement of PI3K/AKT signaling in ICS II-mediated regulation of mitochondrial homeostasis and neuronal survival. Collectively, these findings extend the mechanistic understanding of ICS II-mediated neuroprotection and support its further investigation as a natural product-derived candidate for AD intervention.

## Data Availability

The original contributions presented in the study are publicly available. This data can be found here: https://ngdc.cncb.ac.cn/gsa, accession number CRA04996.
