# Supplementary material for: Icariside II protects against neuronal injury by modulating PI3K/AKT-dependent mitochondrial dynamics and apoptosis in Alzheimer’s disease models
Source: Front Pharmacol. 2026 Sep 21;17:1911518. doi: 10.3389/fphar.2026.1911518 (PMC13635299; doi:10.3389/fphar.2026.1911518)
Supplement: Supplementary file 1 [file DataSheet1.docx]

**Supplementary Methods**

1. Quantitative analysis of Nissl-positive neurons

Blind analysis was performed using ImageJ software: within the above brain regions, neurons with clear cytoplasm, distinct nucleoli, and intact cell bodies (Nissl-positive neurons) were manually counted, avoiding cells with blurred outlines or shrinkage. Nissl-positive neuronal density was expressed as cells/mm² (Cai et al., 2018; Liang et al., 2023; Okada et al., 2019).

2. Preparation of oligomeric Aβ_1-42_

Preparation of oligomeric Aβ_1-42_ was based on previously reported protocols (Wang et al., 2023; Xiao et al., 2020). Briefly, the Aβ_1-42_ peptide was dissolved in hexafluoroisopropanol (HFIP), dried to form a film, and then reconstituted in anhydrous DMSO. The resulting solution was diluted with H-DMEM medium to a final concentration of 200 μM and incubated at 4°C for 24 h to promote oligomer formation, ultimately yielding a solution highly enriched in oligomeric species.

**Abbreviations**

| AD | Alzheimer’s disease |
| --- | --- |
| Aβ | β-amyloid protein |
| AKT | protein kinase B |
| APP | Amyloid precursor protein |
| Bax | Bcl-2-associated X |
| Bcl-2 | B-cell lymphoma-2 |
| Caspase-3 | cysteinyl aspartate specific proteinase-3 |
| CCK-8 | Cell counting kit-8 |
| Cytc | Cytochrome c |
| DEGs | differentially expressed genes |
| Drp1 | dynamin-related protein 1 |
| GO | Gene Ontology |
| GSH | Glutathione |
| ICS II | Icariside II |
| KEGG | Kyoto Encyclopedia of Genes and Genomes |
| LDH | Lactate dehydrogenase |
| MDA | Malondialdehyde |
| Mfn2 | Mitofusin 2 |
| PI3K | phosphatidylinositol-3-kinase |
| ROS | reactive oxygen species |
| SOD | superoxide dismutase |

**Supplementary Figure. 1 Effects of ICSII on viability of normal HT22 cells.**

Reference

CAI, J. C., LIU, W., LU, F., KONG, W. B., ZHOU, X. X., MIAO, P., et al. (2018). Resveratrol attenuates neurological deficit and neuroinflammation following intracerebral hemorrhage. *Exp Ther Med,* 15 (5)**,** 4131–4138. doi: 10.3892/etm.2018.5938

LIANG, T., ZHU, Z., GONG, F., YANG, X., LEI, X. & LU, L. (2023). Galectin-3 promotes brain injury by modulating the phenotype of microglia via binding TLR-4 after intracerebral hemorrhage. Aging (Albany NY), 15 (17), 9041–9058. doi: 10.18632/aging.205014

OKADA, T., ENKHJARGAL, B., TRAVIS, Z. D., OCAK, U., TANG, J., SUZUKI, H., et al. (2019). FGF-2 Attenuates Neuronal Apoptosis via FGFR3/PI3k/Akt Signaling Pathway After Subarachnoid Hemorrhage. *Mol Neurobiol,* 56 (12)**,** 8203–8219. doi: 10.1007/s12035-019-01668-9

WANG, X., XIE, J., TAN, L., LU, Y., SHEN, N., LI, J., et al. (2023). N6-methyladenosine-modified circRIMS2 mediates synaptic and memory impairments by activating GluN2B ubiquitination in Alzheimer's disease. *Transl Neurodegener,* 12 (1)**,** 53. doi: 10.1186/s40035-023-00386-6

XIAO, H., LI, H., SONG, H., KONG, L., YAN, X., LI, Y., et al. (2020). Shenzao jiannao oral liquid, an herbal formula, ameliorates cognitive impairments by rescuing neuronal death and triggering endogenous neurogenesis in AD-like mice induced by a combination of Aβ42 and scopolamine. *J Ethnopharmacol,* 259**,** 112957. doi: 10.1016/j.jep.2020.112957
