# Supplementary material for: Icariside II protects against neuronal injury by modulating PI3K/AKT-dependent mitochondrial dynamics and apoptosis in Alzheimer’s disease models
Source: Front Pharmacol. 2026 Sep 21;17:1911518. doi: 10.3389/fphar.2026.1911518 (PMC13635299; doi:10.3389/fphar.2026.1911518)
Supplement: Supplementary file 2 [file DataSheet2.docx]

**The original images of Western blot experiments**
